# Out of sight of wind turbines—Reindeer response to wind farms in operation

**DOI:** 10.1002/ece3.4476

**Published:** 2018-09-03

**Authors:** Anna Skarin, Per Sandström, Moudud Alam

**Affiliations:** ^1^ Department of Animal Nutrition and Management Swedish University of Agricultural Sciences Uppsala Sweden; ^2^ Department of Forest Resource Management Swedish University of Agricultural Sciences Umeå Sweden; ^3^ Section of Statistics School of Technology and Business Studies Dalarna University Falun Sweden

**Keywords:** anthropogenic disturbance, calving season, cumulative impact, habitat selection, large herbivore, *Rangifer tarandus*, renewable energy, semi‐domesticated reindeer

## Abstract

To meet the expanding land use required for wind energy development, a better understanding of the effects on terrestrial animals’ responses to such development is required. Using GPS‐data from 50 freely ranging female reindeer (*Rangifer tarandus*) in the Malå reindeer herding community, Sweden, we determined reindeer calving sites and estimated reindeer habitat selection using resource selection functions (RSF). RSFs were estimated at both second‐ (selection of home range) and third‐order (selection within home range) scale in relation to environmental variables, wind farm (WF) development phase (before construction, construction, and operation), distance to the WFs and at the second‐order scale whether the wind turbines were in or out of sight of the reindeer. We found that the distance between reindeer calving site and WFs increased during the operation phase, compared to before construction. At both scales of selection, we found a significant decrease in habitat selection of areas in proximity of the WFs, in the same comparison. The results also revealed a shift in home range selection away from habitats where wind turbines became visible toward habitats where the wind turbines were obscured by topography (increase in use by 79% at 5 km). We interpret the reindeer shift in home range selection as an effect of the wind turbines per se. Using topography and land cover information together with the positions of wind turbines could therefore help identify sensitive habitats for reindeer and improve the planning and placement of WFs. In addition, we found that operation phase of these WFs had a stronger adverse impact on reindeer habitat selection than the construction phase. Thus, the continuous running of the wind turbines making a sound both day and night seemed to have disturbed the reindeer more than the sudden sounds and increased human activity during construction work.

## 1 INTRODUCTION

The demand for renewable energy is rapidly increasing and placing an expanding pressure on land use (Northrup & Wittemyer, 2013). Wind power capacity is increasing exponentially across the globe. By 2021, a 12‐fold increase is predicted from today's 60 GW (end of 2017) up to 800 GW (Sawyer, 2017). Thus, there is a rapid expansion of the footprint of wind farms (WFs) with their associated infrastructure of power lines and road networks. WF establishments add to the impact of already on‐going human activities such as roads, forestry, hydropower, and mining. Together, such activities fragment the landscape creating a complex pattern of cumulative impacts (Gillingham, Halseth, Johnson, & Parkes, 2016).

To date most research on environmental impact of WFs, and possible mitigation measures have been developed in relation to avian species, such as sea birds and raptors, and bats (e.g., Thaxter et al., 2017; Warwick‐Evans, Atkinson, Walkington, & Green, 2018; Wiens et al., 2017). Research on how WF developments affect terrestrial animals is increasing. For example, roe deer (*Capreolus capreolus*) and hare (*Lepus europaeus*) in the agricultural landscape (Łopucki & Mróz, 2016), and wolves (*Canis lupus*) in inland mountain regions (Ferrão da Costa, Paula, Petrucci‐Fonseca, & Álvares, 2018) have been shown to be negatively affected by WFs, while common pheasant (*Phasianus colchicus)* seem to be positively affected and red fox (*Vulpes vulpes*) did not react to WF development (Łopucki & Mróz, 2016). However, there is still a pressing need for better understanding of the mechanisms, and potential adverse effects on terrestrial mammals’ responses to WF development and whether responses differ in relation to species and habitat types, to make it possible to allow knowledge based decisions in relation to conservation management (Helldin et al., 2017).

Reindeer and caribou (both *Rangifer tarandus*) are considered keystone species in northern landscapes (Vors & Boyce, 2009), and also the foundation for reindeer husbandry for numerous indigenous people across the circumpolar region (Jernsletten & Klokov, 2002). Over the last century, *Rangifer* habitats have been exposed to major changes due to forestry, mining, hydro power, and other exploitation (Gillingham et al., 2016; Johnson & Russell, 2014; Kivinen, 2015). Recently, this exploitation has been accompanied by the development of WFs. In the reindeer husbandry area in Sweden alone, there are currently 1,013 wind turbines in place, another 1,696 are approved and applications have been submitted for a further 1,838 (www.vindbrukskollen.se, retrieved 30 May 2018). Both wild and domesticated *Rangifer* are known to respond to disturbances with regional‐scale avoidance or decreased use of exploited areas (Skarin & Åhman, 2014; Vistnes & Nellemann, 2008). To date, a few studies have examined the impact of WF construction and operation in relation to semi‐domesticated reindeer behavior response and habitat selection, and these show limited (Colman, Eftestol, Tsegaye, Flydal, & Mysterud, 2012, 2013; Flydal, Eftestøl, Reimers, & Colman, 2004; Tsegaye et al., 2017) to strong negative effects of the WFs (Skarin & Alam, 2017; Skarin, Nellemann, Rönnegård, Sandström, & Lundqvist, 2015; Skarin, Sandström, Alam, Buhot, & Nellemann, 2016). This variation in results can partly be explained by differences in the geographical and the seasonal range of the studies (Skarin & Åhman, 2014), from fine‐scale behavioral studies of fenced reindeer (Flydal et al., 2004) and habitat selection studies at the intermediate scale in summer (Colman et al., 2012, 2013) or all‐year around (Tsegaye et al., 2017) to regional scale during calving and summer seasons in the boreal forests (Skarin & Alam, 2017; Skarin et al., 2015). So far, most research shows that construction phase is more severe than operation phase (Colman et al., 2012, 2013; Tsegaye et al., 2017). However, Skarin and Alam (2017) found indications of operation phase having a larger impact on reindeer regional‐scale habitat selection than construction phase, although a further study of reindeer movement and habitat selection was needed to gain a better understanding of the mechanisms behind this suggested avoidance. Noise from wind turbines appears to disturb animals, hinder their vocal communication, and their ability to hear predators leading to modified habitat use (Rabin, Coss, & Owings, 2006; Shannon et al., 2016). Furthermore, prey animals like reindeer react to movements in their sight (D'Angelo et al., 2008; Heesy, 2004) and may, therefore, react to the movement of the turbine blades. To our knowledge, there is only one study of wind turbine noise and visual cues on free‐living terrestrial animal behavior, performed on ground squirrels (Rabin et al., 2006), and there seem to be no previous studies of possible impacts of sight and sound from WFs on either free‐ranging reindeer or caribou.

Calving is an especially sensitive time period for *Rangifer* (e.g., Wolfe, Griffith, & Wolfe, 2000). The animals tend to search for a calm, predator free environment for themselves and their calf (e.g., Pinard, Dussault, Ouellet, Fortin, & Courtois, 2012). Wild reindeer have been shown to avoid calving close to a road crossing (Panzacchi, Van Moorter, & Strand, 2013); however, they do not seem to avoid power‐line developments (Colman et al., 2015). Semi‐domesticated reindeer's selection of calving sites in relation to anthropogenic development is less well investigated, but there is evidence revealing female reindeer's avoidance of cabins (Skarin, Danell, Bergstrom, & Moen, 2008), WF construction areas (Skarin et al., 2015), power‐line construction (Eftestøl, Tsegaye, Flydal, & Colman, 2015), roads, and power‐lines (Vistnes & Nellemann, 2001) during the calving season. Apart from the human activity within a WF, the noise and visual cues from the wind turbines may disturb reindeer during the sensitive calving season.

The aim of this study was to investigate reindeer selection of calving sites and habitat during the calving season around two small WFs in a boreal forest landscape. We analyzed data from GPS equipped reindeer for the periods before construction, during construction and during operation of the WFs. We combined the GPS location data with knowledge from reindeer herders about the reindeer range use and their herding strategies. We studied reindeer fine‐scale movement to determine calving sites, and we investigated reindeer habitat selection following Johnson's (1980) second‐ (selection of home range) and third‐order (selection within home range) scale of selection, and developed resource selection functions (RSFs) in relation to the WF site, based on information about land cover type, topography, and existing infrastructure (roads and power lines) before and during construction, and during operation.

## 2 METHOD

### 2.1 Study area

**Figure 1:**
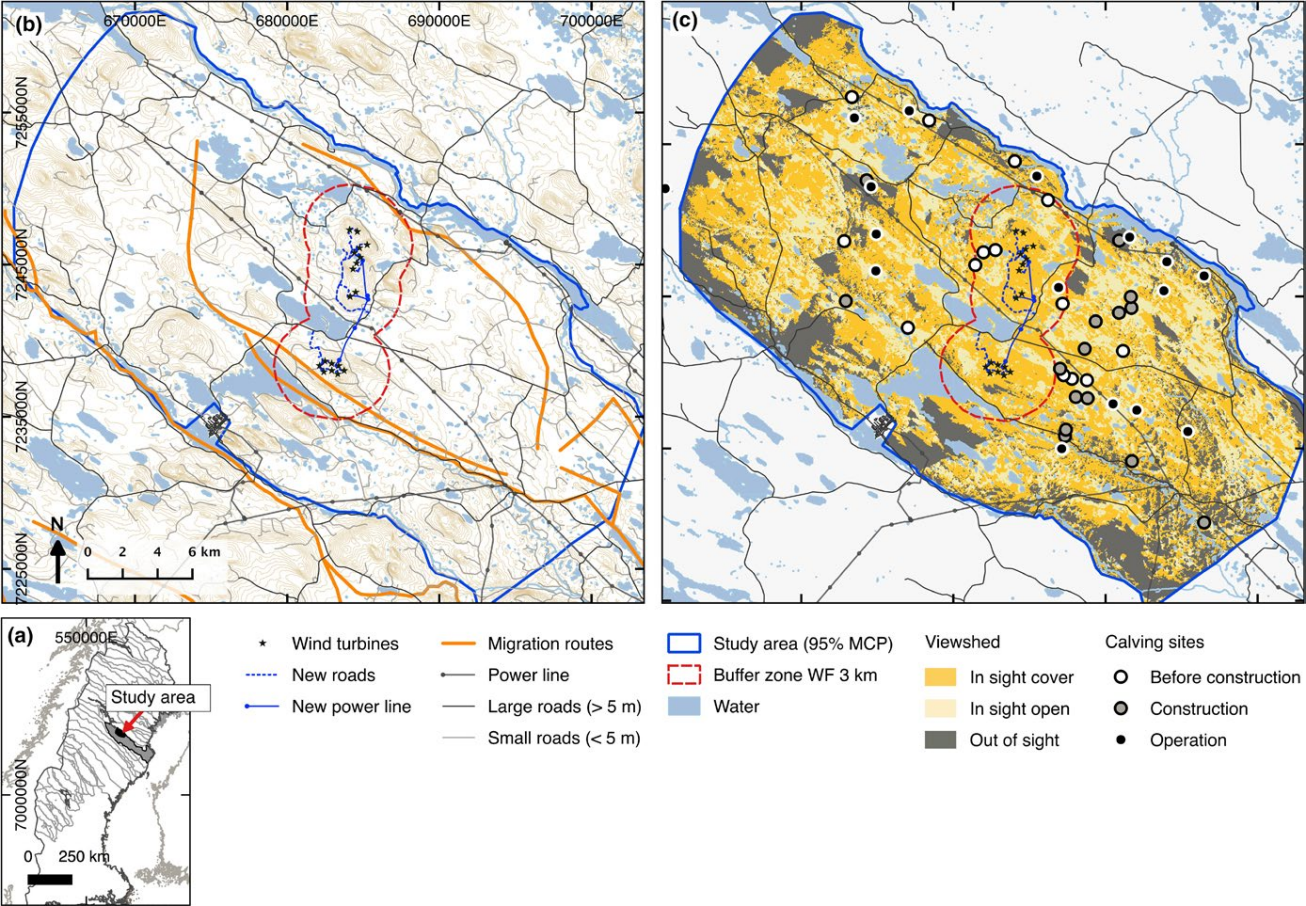
(a) Overview map showing study area position and the borders of the Sami reindeer herding communities in Sweden, (b) map of the Malå reindeer herding community calving range including the wind farm sites and traditional migration routes, the buffer zone of 3 km around the wind farm sites, (c) a background map of wind turbine viewshed in three categories: wind turbines out of sight, in sight with cover and in sight in open areas including identified calving sites for 45 female reindeer before and during construction and during operation. ©Lantmäteriet

In Sweden, reindeer husbandry is carried out in the northern half of the country (Figure 1a) migrating between different seasonal ranges. However, no part of the reindeer husbandry area is set aside exclusively for reindeer husbandry, it is always carried out in conjunction with other land use (Sandström et al., 2003). The study area, 1,350 km^2^ in size, situated in the boreal forest, cover calving, and postcalving ranges of the Malå forest reindeer herding community (65°14′, 18°58′; Figure 1b). Two WFs with 8 and 10 wind turbines (149 m in height), respectively, were constructed on Storliden and Jokkmokksliden mountains in the centre of the calving range. The two WFs were constructed 4 km apart during the years 2010–2011, hereafter referred to as the “construction phase.” For a detailed description of the study area and reindeer use during the construction phase, please refer to Skarin et al. (2015) and Skarin and Alam (2017). The years after construction, 2012 and after when the wind turbines were running, are hereafter referred to as the “operation phase.”

The study area was used by a part of the total herd of the Malå reindeer herding community: approximately 1,200–3,000 (pers. comm. Jan Rannerud and Tomas Stenlund, Malå reindeer herding community) female reindeer and their calves (the total number of female reindeer in the whole reindeer herding community ranged between 4,144, and 4,854 over the study years). Every year in April reindeer herders migrated with the reindeer herd “on foot,” except in 2015 when the reindeer were moved by truck, from the winter ranges in the east to the summer ranges in the west. After migration, from the beginning of May, the reindeer were free ranging and used the calving grounds based on their own habitat preferences. By the end of June, reindeer were gathered and moved by the herders to the closest corral for the yearly calf‐marking event. The study covered the free‐ranging period from the beginning of May until the end of June, during 2008–2009 (before), 2010–2011 (construction phase), and 2015–2016 (operation phase).

### 2.2 GPS data

The analysis was based on GPS positions of adult females fitted with GPS‐collars (Followit Lindesberg AB, reindeer collar), which at some point, approached within 2 km of the WFs during the calving season (Table 1). All females were assumed to be calving during the study period. We did not use information from reindeer with more than 15% missing data, ending up with data from 50 individual females, three of which had collars attached during two calving seasons (2015 and 2016). In total, 28,063 positions, collected at 2 hr intervals, were used for the analysis.

**Table 1:** Study phases in the Malå study area in northern Sweden, over the six study years, with number of days and number of reindeer fitted with devices providing a GPS position every 2 hr

Year	Date	Number of reindeer with GPS‐collar	Wind power development phase
2008	12/5‐18/6	14	Before construction
2009	2/5‐19/6	6	Before construction
2010	10/5‐24/6	13	Construction
2011	10/5‐22/6	3	Construction
2015	1/5‐25/6	10	Operation
2016	5/5‐23/6	8	Operation

### 2.3 Habitat variables

We included habitat variables known or suspected to influence reindeer habitat selection in this area (Skarin et al., 2015). These were land cover type, elevation, slope, minimum distance to water, road, power lines and wind turbines, information on whether the wind turbines were in the viewshed (i.e., if they were visible) or not based on topography and forest cover at each given point. All variables were screened for collinearity using variance inflation factors (VIF; Zuur, Ieno, & Elphick, 2010), with VIF ≥ 3.0 as a threshold for removing a variable. The habitat variables were first extracted using QGIS Desktop. All the digital geographical data were provided by Lantmäteriet (http://www.lantmateriet.se). We used the Swedish Landcover Map (SMD, Naturvårdsverket, 2014), 25 m resolution, describing 43 land cover classes. We complemented the SMD data, which originates from satellite images from 2000, with mapped clear cuts from 2000 to 2016 (Swedish forest agency; https://www.skogsstyrelsen.se/skogligagrunddata) and reclassified it from 43 to 5 classes: forest, young forest, clear cuts, mires, and heath (Table 2). All distance variables (measured in meters) were transformed to exponential decays of the form *e* ^−*α*d^, where d is the distance to the feature and *α* is set to correspond to an approximate effect zone as animals’ response to landscape features probably decreases at greater distances (Nielsen, Cranston, & Stenhouse, 2009). Reindeer avoidances to larger roads and power lines have been shown to decline at around 1–2 km (Anttonen, Kumpula, & Colpaert, 2011; Lundqvist, 2007; Panzacchi, Van Moorter, Jordhoy, & Strand, 2012) why *α* was set to 0.002 (approximate effect zone <1,500 m), for road and power lines. Distance to water never exceeded 1,552 m, but to set all distance variables to the same scale, we calculated decay distance to water with *α *= 0.002. To calculate the minimum distance to the nearest large road, we used roads classified as wider than 5 m (http://www.lantmateriet.se), termed public roads with regular traffic. Similarly, minimum distance to small (forest) roads was calculated for roads classified as narrower than 5 m. For WF, we assumed no variation based on distance; therefore, we analyzed four different decay distances *α* = 0.001, 0.0002, 0.0003 and 0.0005 (approximate effect zone <2,500, <10,000, <7,500 and <5,000 m, respectively) for the model selection setup. These distances were based on earlier analysis of GPS‐data in which reindeer have been shown to have less effective habitat use (shown as an increase in step length) within 5 km of WFs during operation and construction compared to before construction (Skarin et al., 2015, 2016). Exponential decay distances ranged between 1 at the feature to 0 at very great distances. The Digital Elevation Model (DEM) had a 2‐m resolution (Swedish forest agency; https://www.skogsstyrelsen.se/skogligagrunddata). To reduce computational load, the 2‐m DEM grid was resampled to a 25‐m grid using the GRASS (http://grass.osgeo.org) resampling module. The layer depicting whether the wind turbines were in or out of sight based on topography was calculated using the QGIS viewshed analysis (http://hub.qgis.org/projects/viewshed/wiki) plugin. The 25 m DEM together with the wind turbines’ position and their overall height (149 m including the rotor blades) were used for the calculations. The “target height,” the average height for an adult reindeer, was set to 110 cm. Output data for the viewshed analysis were classified as out of sight (hereafter referred to as *out of sight*) and in sight when at least one wind turbine was in sight and not shielded by topography (hereafter referred to as *in sight*). To take into account vegetation coverage, *in sight* areas with land cover classes heath, mires, and clear cuts were classified as *in sight open* areas, and *in sight* areas with land cover classes forest and young forest, were classified as *in sight cover* areas (Figure 1c). The ruggedness index (VRM) was calculated from the 25 m DEM layer as described by Sappington, Longshore, and Thompson (2007) with a 5 × 5 neighborhood. Slope in degrees was calculated from the 25 DEM layer using the “raster” library in R. Finally, to reduce computational load and to make the analysis comparable with the analysis in Skarin et al. (2015), all raster layers were resampled to a 50‐m grid using the nearest neighborhood majority filter for categorical variables and mean filter for continuous variables.

**Table 2:** Mean values and ranges (continuous variables) or percentage (categorical variables) of habitat variables (50‐m resolution) used in the resource selection functions within the Malå study area

Habitat variable	Mean (ranges) or per cent
*Continuous variables*	
Elevation (m)	347 (234–558)
Ruggedness index	0.00039 (0‐0.036)
Slope (degrees)	2.25 (0–32.9)
Distance to roads (m)	1030 (0–4681)
Distance to wind turbines (m)	10390 (50–22241)
Distance to power lines (m)	3040 (0–12164)
Distance to water (m)	306 (0–1552)
*Viewshed*	
In sight cover	45%
In sight open	33%
Out of sight	22%
*Land cover class*	*2008–2011*—*2015–2016*
Heath	1%—1%
Forest	40%—38%
Clear	12%—8%
Young	20%—26%
Mire	27%—27%

*Notes*

Land cover classes changed in proportions between the study period 2008–2011 and 2015–2016, as new clear cuts appeared in the area and old clear cuts became young forest and young forest became forest.

### 2.4 Analysis of calving site

Calving starts at the beginning of May and lasts until the beginning of June, with peak calving occurring in mid‐May (Eloranta & Nieminen, 1986; Panzacchi et al., 2013). We estimated specific calving sites for each female for this time period quantifying the time (i.e., number of locations) spent within a patch of a given radius, using the residence Time‐function from the “adehabitatLT”‐library (Barraquand & Benhamou, 2008; Calenge, Dray, & Royer‐Carenzi, 2009) in the R software (R Core Team 2017), similar to Panzacchi et al. (2013). We examined results based on radii of 50, 100, 150, 200, 250, 300, and 350 m. Once the optimal radius was determined we used the lavielle function, within the same library, to perform a nonparametric segmentation of the movement trajectory. This allowed us to manually select the segment representing the positions with a peak in residence time. If more than one peak was present, we selected the segment with the highest peak (Panzacchi et al., 2013), and if there were two peaks of equal height, we selected the first peak in time. Before parturition, it is less likely that a female will stay longer at single patches, while the mother and calf pair move slowly to begin with (Espmark, 1971a), and may stay longer in single patches, which are not necessarily the same as the birth patch. We estimated the mean position from each identifiable calving site and calculated the minimum distance to the nearest wind turbine. Comparisons of change in the average minimum distance to the nearest wind turbines between before and during construction, and before construction and during operation were tested using a Wilcoxon Mann‐Whitney test.

### 2.5 Analysis of habitat selection

Resource selection functions models can provide estimates of animal selection of habitats at different scales (Johnson, 1980; Johnson, Nielson, Merrill, McDonald, & Boyce, 2006). We developed RSF models with a use‐availability design, using binomial family generalized linear mixed models, evaluating whether the WFs affected reindeer habitat selection at Johnson's (1980) second‐ and third‐order scales before and during construction and operation phases (Skarin et al., 2015). A random intercept for each individual was estimated at both scales of selection, to account for possible individual variation in habitat selection (Gillies et al., 2006). To assess the second‐order scale (i.e., selection of home range), we compared habitat variables at reindeer GPS locations to random locations within the calving range. We defined the calving range by computing the 95% (to exclude outliers) minimum convex polygon of all GPS‐positions and using the boundaries of the reindeer herding community (Figure 1b). For this we used 26,445 GPS‐locations, as 1,618 locations fell outside of the defined calving range. At the third‐order scale (i.e., selection within the home range), we compared habitat variables at reindeer GPS locations to random locations within the individual Brownian Bridge Movement Model (BBMM) home ranges (Horne, Garton, Krone, & Lewis, 2007). The Kernelbb‐function from the “adehabitatHR” (Calenge et al., 2009) library in R was used for the home range estimation. We estimated the utilization distribution (UD) for each individual using a Brownian bridge kernel method (Calenge, 2006; Horne et al., 2007). The spatial extent of the UD was defined as the 99% BBMM home range boundary and was displayed on a 50‐m grid. We used an estimated location error of 20 m. For the RSF‐model we used 26,794 GPS‐locations inside the defined BBMMs. At both scales, we generated available points using a 1:1 ratio of used to available locations. We split the locations based on the WF development phases and allowed for an interaction between decay distance (using the four different alternatives of decay in the model selection procedure; Supporting information Appendix 1: Figure S1, Table S1, S2) to the wind turbines and class of viewshed or land cover class (two alternatives in the model selection procedure). This interaction between GPS locations and the three variables made it possible to assess whether the reindeer changed habitat selection in relation to the WFs. To allow the models to converge, we standardized elevation, slope, and ruggedness (by shifting the centre to their means, and scaling with the respective standard deviation). AIC‐values were used to identify the most parsimonious model. To illustrate the results from the RSF models, we calculated predicted probabilities of selection and the 95% confidence intervals (CI) to show the marginal effects of the variable. Thus, the predicted probabilities for a given predictor variable were calculated while keeping the other predictor variables constant (at their mean values, for continuous predictors). In addition, we produced maps showing the predicted probability for each 50 m pixel over the study area calculated from the population‐averaged estimates of the RSFs within each study phase. We subtracted the predicted values for construction and operation phase, respectively, with the predicted values from before construction, thus revealing the change in predicted habitat selection for each phase at both scales of selection. To validate the models with the best fit, we used a k‐fold crossvalidation (Boyce, Vernier, Nielsen, & Schmiegelow, 2002). The predicted probability was arbitrarily divided into ten equal bins. A testing ratio of 20% was determined, and a *k*‐fold partition of five groups was used. This resulted in five correlations to evaluate the model fit.

## 3 RESULTS

### 3.1 Calving sites

We identified 45 calving sites based on GPS‐data from 50 individuals. For eight potential calving events, we could not identify any clear pattern of calving site, indicating either that these females did not have a calf or that the movement pattern was not consistent enough to identify the event. The best search radius to identify calving sites was 200 m. The mean time the female spent at the site was 56 hr (*SD* = ±26; range 18–138 hr). Comparing distance to WFs during the different activity phases, we found a significant increase in distance between calving sites and the nearest wind turbine during the operation phase (median distance = 9,153 m, 95% CI = ±2,511, *N* = 16) compared to the before construction phase (median = 4,222 m, 95% CI = ±2,137, *N* = 14; Wilcoxon Mann‐Whitney *p*‐value = 0.02). However, the mean distance to wind turbines during the construction phase (median = 5,552 m, 95% CI = ±1,834, *N* = 15) did not vary significantly from before construction (Wilcoxon Mann‐Whitney *p*‐value = 0.07). We detected one calving event within 3 km of the WFs during operation, compared to five events before construction (Figure 1c).

### 3.2 Habitat selection

Among the habitat variables, the VIF did not indicate any apparent multicollinearity (VIF < 2) except for viewshed (VIF = 7.3 and 16.6, home range selection and selection within home range, respectively) and land cover (VIF = 7.7 and 15.6). Variables identified in the most parsimonious model in selection of home range were elevation, slope, decay distance to large road, power lines and water, and decay distance to wind turbines interacting with study phase and viewshed class (Supporting information Table S1). At the home range scale selection, the decay factor of 0.0002 in relation to distance to wind turbines resulted in the most parsimonious model. At selection within the BBMM home range variables identified for the most parsimonious model were elevation, ruggedness, decay distance to power lines, water and large and small roads, and decay distance to wind turbines interacting with study phase and land cover class. The decay factor of 0.0005 gave a better model fit compared to the other decay factors (Supporting information Table S2). This indicates that the effect of the distance to the WFs virtually vanishes at approximately 10 km in the selection of home range compared to 5 km in the selection within the home range.

**Figure 2:**
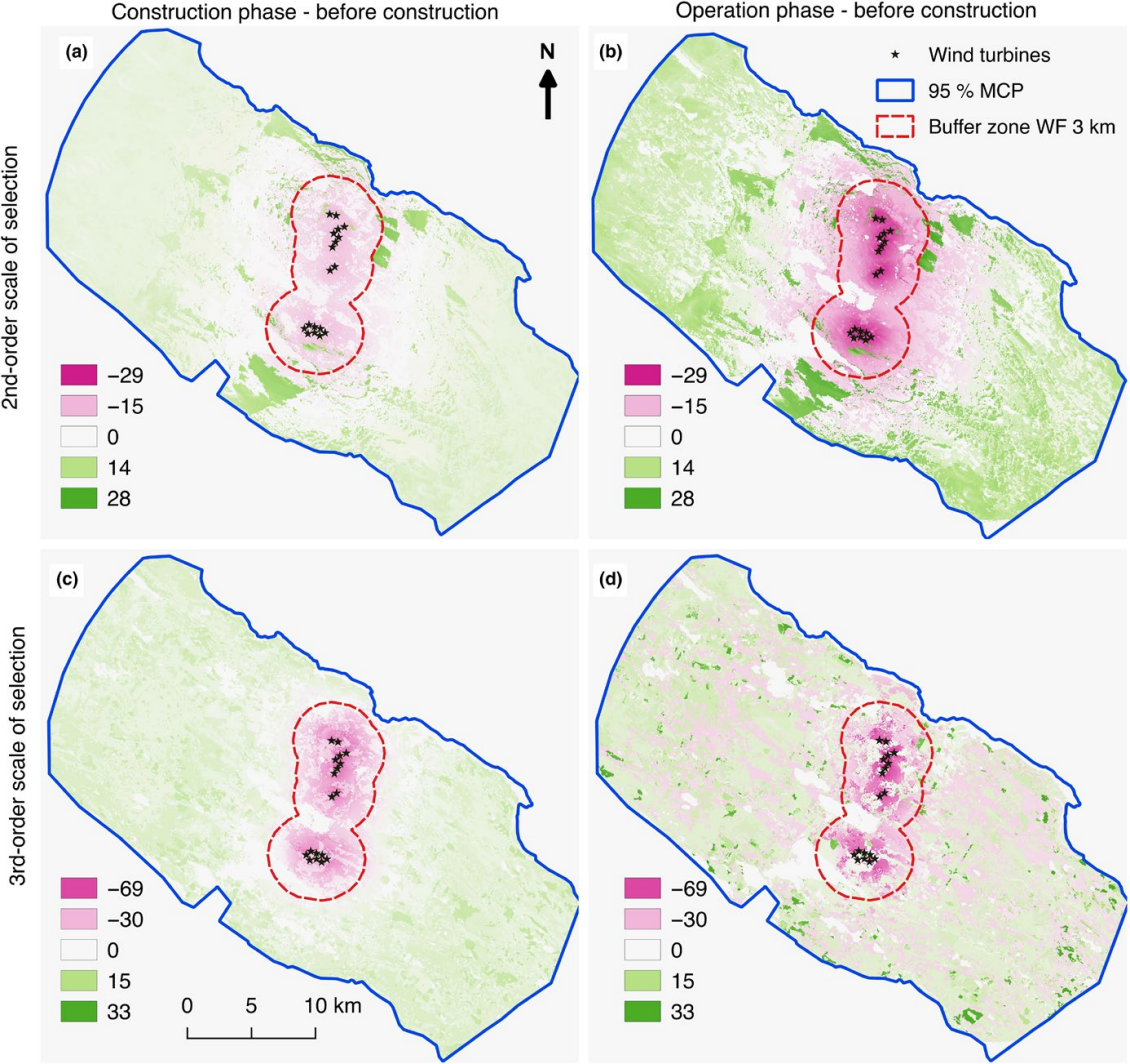
Maps showing the difference in predicted habitat selection (green—increase in selection, pink—decrease in selection, and white—no change) from estimated resource selection functions between (a) construction phase and before construction, (b) operation phase and before construction at the second‐order scale (i.e., home range selection), (c) construction phase and before constriction, and (d) operation phase and before construction at the third‐order scale (i.e., selection within home range) in the Malå reindeer herding community during the calving season

**Figure 3:**
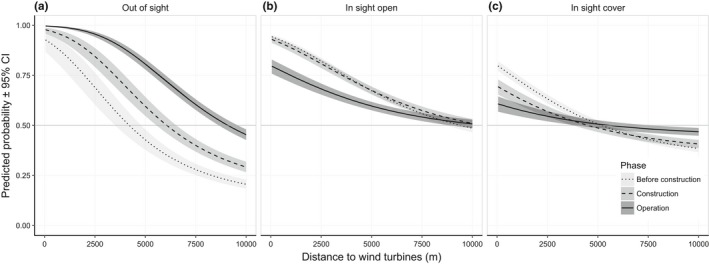
Marginal predicted probability (±95% CI) of reindeer presence at the second‐order scale (i.e., home range selection), for (a) out of sight, (b) in sight open, and (c) in sight cover areas, in relation to distance to the wind turbines and the wind farms’ development phases (before construction, construction, and operation) from the RSF‐models, in the Malå reindeer herding community during the calving season

At both scales of selection (Tables 3 and 4), reindeer preferred the WF sites and the surrounding areas before construction and then decreased their use of these areas both during construction (as already reported in Skarin et al., 2015) and during operation phase. This produced a different pattern in the habitat selection of the calving range compared to the situation before construction (Figure 2 and Supporting information Figure S1). In selection of the home ranges before construction, the reindeer preferred areas where the constructed wind turbines later would be in sight and then switched to preferring areas where the wind turbines were out of sight during the operation phase (Figure 3). Predicting the marginal effect of distance from the wind turbines in interaction with viewshed class and phase, there was a 14% increase in selection of out of sight areas at 1 km from the wind turbines during the operation phase compared to before construction and a 79% increase in out of sight areas at 5 km (Figure 2b and 3a). Correspondingly, selection of in sight open areas decreased by 17% at 1 km and 13% at 5 km (Figure 3b), and selection of in sight cover decreased by 22% at 1 km and at 5 km from the WFs no change was detected (Figure 3c). During the construction phase, the selection of out of sight areas also increased compared to before construction, but not to the same extent as during the operation phase (e.g., at 1 km it increased by 9% and at 5 km by 37%). Selection of in sight cover areas decreased almost to the same extent in the construction phase as in the operation phase. There was no change in the selection of in sight open areas between the construction phase and before construction.

**Table 3:** Estimates of resource selection function models of the second‐order scale, that is, selection home range, for female reindeer in and around the wind farm sites before (2008–2009) and during construction (2010–2011), and during operation (2015–2016) in the Malå reindeer herding community calving ranges

	Estimate	Std. Error	Pr(>|z|)
(Intercept)	−0.485	0.052	<0.001
Decay distance to WF^a^	2.152	0.094	<0.001
Decay distance to water^b^	−0.446	0.043	<0.001
Decay distance to power lines^b^	0.422	0.041	<0.001
Decay distance to roads^b^	−0.424	0.035	<0.001
Elevation (m)	−0.101	0.011	<0.001
Slope (degrees)	−0.207	0.011	<0.001
Phase			
Construction phase	0.198	0.068	0.004
Operation phase	0.545	0.064	<0.001
View shed class			
In sight open	0.251	0.054	<0.001
Out of sight	−1.202	0.099	<0.001
Interactions			
Construction: In sight open	−0.048	0.080	0.546
Operation: In sight open	−0.214	0.075	0.004
Construction: Out of sight	0.139	0.136	0.308
Operation: Out of sight	0.294	0.125	0.018
Construction: Decay distance to WF	−0.758	0.144	<0.001
Operation: Decay distance to WF	−1.492	0.134	<0.001
Out of sight: Decay distance to WF	1.205	0.152	<0.001
Out of sight: Decay distance to WF	2.400	0.417	<0.001
Construction: In sight open: Decay distance to WF	0.371	0.242	0.125
Operation: In sight open: Decay distance to WF	−0.308	0.224	0.168
Construction: Out of sight: Decay distance to WF	1.683	0.580	<0.001
Operation: Out of sight: Decay distance to WF	3.879	0.538	<0.001

*Notes*

^a^Minimum distance in meters to the nearest wind turbine transformed to decayed distance using = exp(−2e‐04 ×  distance) for second‐order scale of selection. ^b^Minimum distance in meters to the nearest road (>5 m wide), power line, and watercourse transformed to decayed distance using exp(−0.002 ×  distance).

**Table 4:** Estimates of resource selection function models of the third‐order scale, that is, selection within home range for female reindeer in and around the wind farm sites before (2008–2009) and during construction (2010–2011), and during operation (2015–2016) in the Malå reindeer herding community calving ranges

	Estimate	Std. Error	Pr(>|z|)
(Intercept)	−0.751	0.069	<0.001
Decay distance to WF^a^	−1.242	0.166	<0.001
Decay distance to water^b^	0.579	0.042	<0.001
Decay distance to power lines^b^	0.660	0.042	<0.001
Decay distance to roads (>5 m)^b^	−0.107	0.036	0.003
Decay distance to roads (<5 m)^b^	0.274	0.037	<0.001
Elevation (m)	0.268	0.013	<0.001
Ruggedness index	−0.026	0.010	0.008
Land cover type			
Heath	1.121	0.127	<0.001
Clear cut	0.788	0.061	<0.001
Young forest	0.219	0.056	<0.001
Mire	0.161	0.046	0.001
Phase			
Construction phase	0.255	0.090	0.005
Operation phase	0.227	0.088	0.010
Interactions			
Construction: Heath	−0.106	0.201	0.597
Construction: Clear cut	0.198	0.086	0.022
Construction: Young forest	−0.120	0.080	0.134
Construction: Mire	−0.111	0.066	0.094
Operation: Heath	−0.479	0.181	0.008
Operation: Clear cut	0.373	0.091	<0.001
Operation: Young forest	0.129	0.073	0.076
Operation: Mire	−0.607	0.065	<0.001
Heath: Decay distance to WF	1.780	0.632	0.005
Clear cut: Decay distance to WF	0.890	0.292	0.002
Young forest: Decay distance to WF	0.541	0.214	0.011
Mire: Decay distance to WF	1.413	0.241	<0.001
Construction: Decay distance to WF	−1.166	0.311	<0.001
Operation: Decay distance to WF	−0.198	0.253	0.434
Construction: Heath: Decay distance to WF	−0.918	1.495	0.539
Construction: Clear cut: Decay distance to WF	−0.676	0.504	0.180
Construction: Young forest: Decay distance to WF	−0.604	0.426	0.157
Construction: Mire: Decay distance to WF	−0.845	0.472	0.073
Operation: Heath: Decay distance to WF	1.632	1.290	0.206
Operation: Clear cut: Decay distance to WF	−1.628	0.503	0.001
Operation: Young forest: Decay distance to WF	−2.868	0.356	<0.001
Operation: Mire: Decay distance to WF	−0.001	0.412	0.999

*Notes*

^a^Minimum distance in meters to the nearest wind turbine transformed to decayed distance using = exp(‐5e‐04 ×  distance) for third‐order scale of selection. ^b^Minimum distance in meters to the nearest road (>5 m wide), power line, and watercourse transformed to decayed distance using exp(−0.002 ×  distance).

**Figure 4:**
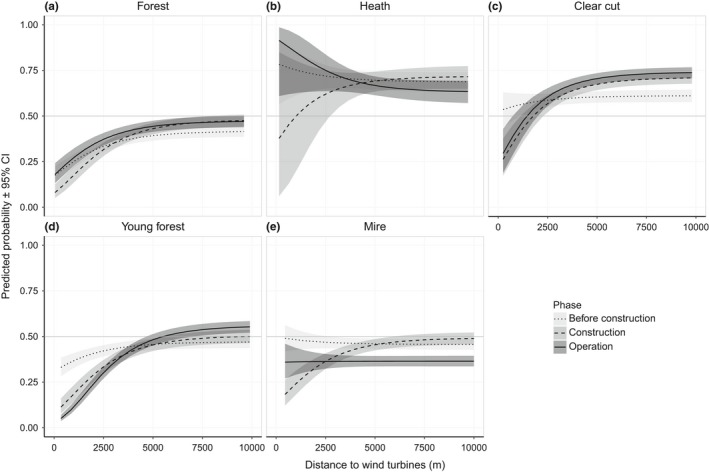
Marginal predicted probability (±95% CI) of reindeer presence at the third‐order scale (i.e., selection within home range), in (a) forest, (b) heath, (c) clear cuts, (d) young forests and (e) mires in relation to distance to the wind turbines and the wind farms’ development phases (before construction, construction, and operation) from the RSF‐model in the Malå reindeer herding community during the calving season

Within the home range, the reindeer preferred heaths and clear cuts over young forest, mires, and forests. Within clear cuts, young forest and mires the reindeer decreased use close to the WFs during both the operation and construction phase compared to before construction (Figure 4). In clear cuts and young forests, the decrease was apparent up to 1 km (Figure 4c) and 3 km (Figure 4d) from the WFs, respectively, with a decrease in use of clear cuts by 29% at 1 km and in young forests by 74% at 1 km and 28% at 3 km. In mires, there was an overall decrease by around 25% in the use of mires during the operation phase compared to before construction, while during the construction phase there was an apparent decline in selection of mires up to 3 km from the WFs (Figure 4e). The reindeer avoided forested areas up to 4 km from the WFs during all study phases. In heaths, we did not find any significant change in selection between the study phases.

Furthermore, at both scales of selection, the reindeer preferred areas close to the power lines, while they avoided areas close to large roads, and steep slopes were avoided in selection of home range and rugged terrain within the home ranges. The reindeer avoided the highest elevations in selection of the home ranges but still preferred the higher elevations within the home ranges. Similarly, they avoided water bodies in the selection of home ranges, while they selected areas closer to these within the home ranges.

The mean Spearman rank for the *k*‐fold crossvalidation at of the RSF‐model representing the selection of home ranges was *r* = 0.951 (*p* < 0.001) and the RSF‐model representing selection within the home range was *r* = 0.964 (*p* < 0.001) for the most parsimonious models found during the study period.

## 4 DISCUSSION

The establishment of the two small WFs in the calving range clearly changed reindeer habitat selection. Before construction, the lowlands (mires, clear cuts, and forest land) east and west of the two mountains where the WFs were established were the most important area for reindeer during the calving season (Skarin et al., 2015). Specific changes in space use during the operation phase include: displacement of calving sites away from WFs, a significant decrease in selecting habitats in areas in proximity to the WFs, and a shift in selection of home ranges where wind turbines became visible towards areas where the wind turbines were obscured by topography. In addition, the operation phase of these WFs had a stronger adverse impact on reindeer habitat selection than the construction phase.

Female reindeer are especially sensitive to disturbance and predation during parturition and the following bonding period (Espmark, 1971a; Pinard et al., 2012). This is when the calf learns to follow the mother based on visual cues, smell, and vocalization, allowing the mother‐calf pair to recognize each other (Espmark, 1971a,1971b). Our results indicate that reindeer selected calving sites further away from the WF area during the operation phase compared to before construction. This could be a consequence of sight and sound from wind turbines disturbing the females in the weeks around parturition. Sound from wind turbines consists of both high– and low‐frequency sound, and the latter may carry over longer distances (van Kamp & van den Berg, 2017). Humans may recognize sound from wind turbines at 1,500 m (Maffei et al., 2015), and depending on sensitivity, it can be experienced as disturbing at 1,000 m or more (van Kamp & van den Berg, 2017; Pierpont, 2009). Reindeer hearing range, tested in a laboratory environment, is similar to human hearing range (Flydal, Hermansen, Enger, & Reimers, 2001); however, we suspect that their sense of hearing is more developed than human hearing and adapted to identify predators through the “normal” background sounds in the natural environment. Increased noise levels in their environment may have an effect on their ability to hear predators, affecting their anti‐predator behavior (Ciuti et al., 2012; Shannon et al., 2016), and it may adversely affect communication between the female and her calf (cf. Rabin et al., 2006). In addition, prey animals like reindeer react to movements in their sight and might move away from moving objects as a strategy to avoid the risk of predation (D'Angelo et al., 2008; Heesy, 2004). Open areas are generally considered the preferred locations chosen by prey animals to allow them to scan for predators (e.g., Altendorf, Laundré, Gonzalez, & Brown, 2001), which was also realized in the preference of heaths and clear cuts within the home ranges. Thus, the importance of the out of sight areas after WF development in selection of home ranges (Figures 2b and 3a) might be explained by the reindeer escaping areas where they could hear the noise of the WFs (Biedenweg, Parsons, Fleming, & Blumstein, 2011; Ciuti et al., 2012; Shannon et al., 2016) and visual disturbance of the movement of the rotor blades in view of the animal's wide‐angle vision (Heesy, 2004). The reduction in the use of mires and open areas in sight of the WFs over large distances (>5 km) in the selection of home ranges (Figure 3c) compared to areas with cover (Figure 3b) suggests that the effect of the visual disturbance dominated further away, while the effect of both sight and sound were evident closer to the WFs. In open areas, the effect of the noise could also have been greater (i.e., the forest is not present to block the sounds of the turbines).

We could not follow changes in individual reindeer selection of calving sites for the phases before and after construction as the GPS‐collar were placed on new individuals each year. A late start of the spring and snow staying longer on the ground in the calving ranges could also cause change of home range selection during the calving season (Sivertsen, 2017). However, the snow disappeared (Malå‐Brännan (Lat—65.1808, Long—18.7431) meteorological station, http://www.smhi.se) within a range of 6 days, comparing before construction (5 May 2008 and 2 May 2009) and the operation phase (30 April 2015 and 1 May 2016). Thus, snow cover most likely did not cause any apparent variation in selection of calving sites or habitat. In addition, results from a parallel study found that pellet‐group counts decreased near the WFs (Skarin & Alam, 2017) supporting the idea of the WFs causing calving‐site displacement. Pellet‐group counts record the use of all animals within the herd, in comparison with GPS‐collars tracking randomly chosen individuals of a whole population. The Rivière‐aux‐Feuilles caribou herd in Labrador, Canada moved their calving ground over 300 km during a period of 15 years (Taillon, Brodeur, Festa‐Bianchet, & Cote, 2012), the reasons for this large displacement were unknown, but it implies that the species is flexible and may find new calving sites if they encounter issues when using the old calving grounds. Such large displacements are not possible within our study area because the reindeer herding communities are limited to their defined and delineated outer boundaries. If the WFs cause females to move out of their usual calving range, it leads to higher densities of reindeer in other parts of the herding community (or in neighboring communities if they accidently move out of the community), and grazing pressure in these areas increases. Shifts in habitat use can also force changes in the overall herding strategies, as the gathering sites close to the WF area will be used less. One of the measures to mitigate the construction of the WFs was to build a new calf‐marking corral west of Jokkmokksliden below the mountain. This new corral has only been used on a few occasions since it was constructed (personal communication, Jörgen Stenberg, Malå community). Thus, selection of calving sites further away from the WFs seems to have resulted in this calf‐marking corral not being used, loosing part of its value as a mitigation measure.

The model selection process indicated that different parameters were important at different scales of selection (e.g., Mayor, Schaefer, Schneider, & Mahoney, 2009; Senft et al., 1987). Landscape characteristics allowing the reindeer to avoid the physical stressor of the WFs’ sound and sight were important in location of the home range, while selection of specific land cover types were important within the home ranges selected, thus following the framework of hierarchical foraging suggested by Senft et al. (1987). This result emphasizes the importance of studying several scales of selection to reach a better understanding of reindeer response to disturbances (Skarin & Åhman, 2014). Construction work of WFs has earlier been suggested to cause more disturbances to reindeer habitat selection than the operation phase of wind turbines (Colman et al., 2012, 2013; Tsegaye et al., 2017), while our results suggest the opposite. During construction work, reindeer migration and movement routes over the main road in the area was cutoff (Skarin et al., 2015). This movement seemed to have been resumed during the operation phase, most likely partly due to that traffic along the roads used for transport of material to the WFs was back to normal levels compared to during construction (Supporting information Figure S1b,c), but still the displacement of the calving sites and the shift in use to the areas out of sight of the WFs were greater. None of the earlier study on WFs and reindeer has investigated the effect of possible sight or sound of wind turbines in relation to change in habitat selection. Colman et al. (2013) and Tsegaye et al. (2017) investigated reindeer habitat selection in relation to distance to the wind turbines both before and after construction, on a peninsula and an island, respectively, and found negative effects of the WFs’ construction, but not of operation of the WFs. In addition, Flydal et al. (2004) compared local behavioral responses of three to five fenced‐in reindeer 0–450 m from a wind turbine with a similar control group 3 km away, but found no systematic difference in behavior. The common feature of the locations of these studies was the limited availability of alternative grazing areas for the reindeer to move away from the WFs. Hence, neither the reindeer regional‐scale response (Skarin & Åhman, 2014; Vistnes & Nellemann, 2008) toward the wind energy development, nor the use of areas where wind turbines were out of sight was possible to measure and evaluate in the experimental designs chosen. This probably explains large parts of the different response pattern found in reindeer in our study.

In conclusion, we interpret the reindeer change of calving sites and shift in use away from habitats where wind turbines where not obscured by topography as an effect of the wind turbines per se. The continuous running of the wind turbines making a sound both day and night seemed to have disturbed the reindeer in our study area more than the sudden sounds and increased human activity during construction work, and as they had the possibility to move away from the WFs this caused significant changes in location of reindeer calving sites and habitat selection. Measurements and evaluation of the spread of the noise from wind turbines and of the effects of visual disturbances from rotor blades need to be evaluated further to fully understand the mechanism behind our findings. Using accurate information describing topography and land cover together with the positions of wind turbines could help identify sensitive habitats for reindeer and improve the planning and placement of wind turbines in reindeer habitats.

If WFs are planned in reindeer habitats, although effects on reindeer habitat selection might be substantial, mitigation measures in relation to reindeer husbandry need to be carefully planned. For example, although the new calf‐marking corral was planned together with the reindeer herding community, in the end it had limited value because the WFs made the reindeer move out and away from this area. Better mitigation efforts to decrease the cumulative pressure could have included construction of well‐functioning wildlife overpasses as well as improvement of forest conditions to facilitate reindeer crossing main roads and movement through the WF areas.

## CONFLICT OF INTEREST

None declared.

## AUTHOR CONTRIBUTIONS

All authors conceived the ideas and designed the methodology; AS collected the data; AS and MA analyzed the data; AS led the writing of the manuscript. All authors contributed critically to the drafts and gave final approval for publication.

## DATA

Data available from the Dryad Digital Repository: https://doi.org/10.5061/dryad.5337hv7.
