# Supplementary material for: Out of sight of wind turbines—Reindeer response to wind farms in operation
Source: Ecol Evol. 2018 Sep 3;8(19):9906–19. doi: 10.1002/ece3.4476 (PMC6202756; doi:10.1002/ece3.4476)
Supplement: Supplementary file 1 [file ECE3-8-9906-s001.pdf]

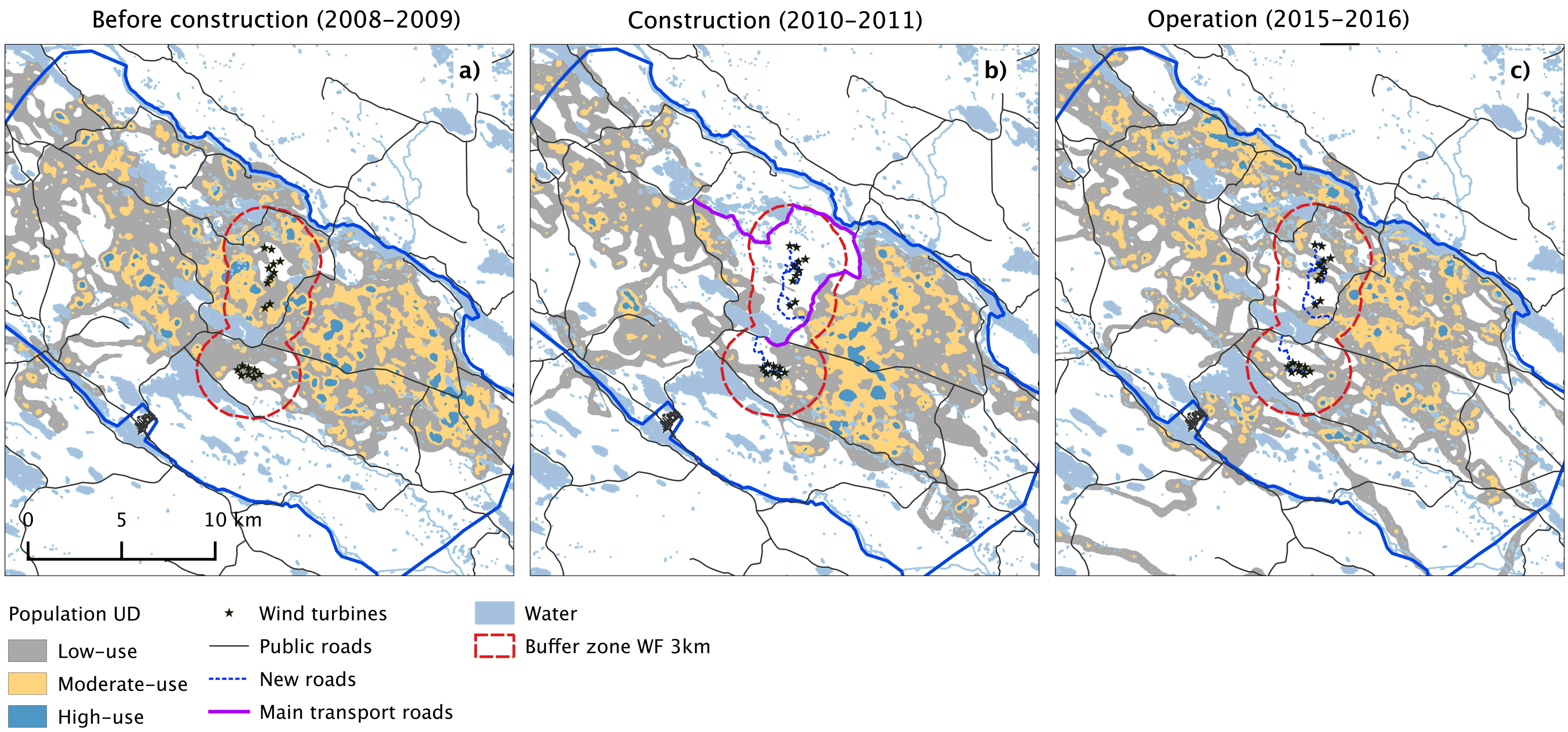
Before construction (2008–2009)
Construction (2010–2011)
Operation (2015–2016)
a)
b)
c)
0
5
10 km
Population UD
Low–use
Moderate–use
High–use
Wind turbines
Public roads
New roads
Main transport roads
Water
Buffer zone WF 3km
