# Supplementary material for: Out of sight of wind turbines—Reindeer response to wind farms in operation
Source: Ecol Evol. 2018 Sep 3;8(19):9906–19. doi: 10.1002/ece3.4476 (PMC6202756; doi:10.1002/ece3.4476)
Supplement: Supplementary file 2 [file ECE3-8-9906-s002.docx]

Table S1. Candidate models for resource selection function estimation at the second-order scale of selection (i.e. selection of home range)

|  | **Candidate models 2nd order** | AIC | ∆AIC | df |
| --- | --- | --- | --- | --- |
| Model 1^a^ | slope + lcover + phase*exp(-0.0005*wind) + exp(-0.002*water) + exp(-0.002*powerl) + exp(-0.002*lroad) | 69812 | 1720 | 15 |
|  | *Elevation and slope vs elevation and ruggedness* |  |  |  |
| Model 2 | dem + slope + lcover +phase*exp(-0.0005*wind) + exp(-0.002*water) + exp(-0.002*powerl) + exp(-0.002*lroad) | 69766 | 1675 | 16 |
| Model 3 | dem + vrm + lcover +phase*exp(-0.0005*wind) + (exp(-0.002*water)+ exp(-0.002*powerl) + exp(-0.002*lroad) | 69870 | 1778 | 16 |
|  | *Viewshed vs land cover* |  |  |  |
| Model 4 | dem + slope + phase*viewshed*exp(-0.0005*wind) + exp(-0.002*water) + exp(-0.002*powerl) + exp(-0.002*lroad) | 69697 | 1606 | 24 |
| Model 5 | dem + slope + phase*lcover*exp(-0.0005*wind) + exp(-0.002*dist) + exp(-0.002*powerl) + exp(-0.002*lroad) | 69287 | 1196 | 36 |
|  | *Decay index 0.001, 0.0003 and 0.0002* |  |  |  |
| Model 6 | dem + slope + phase*viewshed*exp(-0.001*wind) + exp(-0.002*water) + exp(-0.002*powerl) + exp(-0.002*lroad) | 70180 | 2088 | 24 |
| Model 7 | dem + slope + phase*viewshed*exp(-0.0003*wind) + exp(-0.002*water) + exp(-0.002*powerl) + exp(-0.002*lroad) | 68868 | 776 | 24 |
| Model 8 | dem + slope + phase*viewshed*exp(-0.0002*wind) + exp(-0.002*water) + exp(-0.002*powerl) + exp(-0.002*lroad) | 68092 | 0 | 24 |

Note: lcover, land cover class; phase, wind farm development phase; wind, distance to wind turbine; powerl, distance to power lines; lroad, distance to large road; dem, digital elevation model; vrm, vector ruggedness index.

^a^ Start model, same model as the final model for second-order scale selection Skarin et al. (2015) estimating the resource selection function before and during construction phases.

Table S2. Candidate models for resource selection function estimation at the third-order scale of selection (i.e. selection within the BBMM home range)

|  | **Candidate models 3rd order** | AIC | ∆AIC | df |
| --- | --- | --- | --- | --- |
| Model 1^a^ | slope + lcover + phase*exp(-0.0005*wind) + exp(-0.002*water) + exp(-0.002*powerl) + exp(-0.002*lroad) + exp(-0.002*sroad) | 71839 | 793 | 16 |
|  | *Elevation and slope vs elevation and ruggedness* |  |  |  |
| Model 2 | dem + slope + lcover +phase*exp(-0.0005*wind) + exp(-0.002*water) + exp(-0.002*powerl) + exp(-0.002*lroad) + exp(-0.002*sroad) | 71454 | 408 | 17 |
| Model 3 | dem + vrm + lcover +phase*exp(-0.0005*wind) + exp(-0.002*water) + exp(-0.002*powerl) + exp(-0.002*lroad) + exp(-0.002*sroad) | 71451 | 405 | 17 |
|  | *Viewshed vs land cover* |  |  |  |
| Model 4 | dem + vrm + phase*viewshed*exp(-0.0005*wind) + exp(-0.002*water) + exp(-0.002*powerl) + exp(-0.002*lroad) + exp(-0.002*sroad) | 72199 | 1153 | 25 |
| Model 5 | dem + vrm + phase*lcover*exp(-0.0005*wind) + exp(-0.002*water) + exp(-0.002*powerl) + exp(-0.002*lroad) + exp(-0.002*sroad) | 71046 | 0 | 37 |
|  | *Decay index 0.001 and 0.0003* |  |  |  |
| Model 6 | dem + vrm + phase*lcover*exp(-0.001*wind) + exp(-0.002*water) + exp(-0.002*powerl) + exp(-0.002*lroad) + exp(-0.002*sroad) | 71077 | 31 | 37 |
| Model 7 | dem + vrm + phase*lcover*exp(-0.0002*wind) + exp(-0.002*water) + exp(-0.002*powerl) + exp(-0.002*lroad) + exp(-0.002*sroad) | 71337 | 291 |  |
| Model 8 | dem + vrm + phase*lcover*exp(-0.0003*wind) + exp(-0.002*water) + exp(-0.002*powerl) + exp(-0.002*lroad) + exp(-0.002*sroad) | 71198 | 152 | 37 |

Note: lcover, land cover class; phase, wind farm development phase; wind, distance to wind turbine; powerl, distance to power lines; lroad, distance to large road; sroad, small road; dem, digital elevation model; vrm, vector ruggedness index.

^a^ Start model, same model as the final model for third-order scale selection Skarin et al. (2015) estimating the resource selection function before and during construction phases.
